# Tetrodotoxin Sensitivity of the Vertebrate Cardiac Na^+^ Current

**DOI:** 10.3390/md9112409

**Published:** 2011-11-21

**Authors:** Matti Vornanen, Minna Hassinen, Jaakko Haverinen

**Affiliations:** Department of Biology, University of Eastern Finland, Joensuu 80101, Finland; E-Mails: minna.hassinen@uef.fi (M.H.); jaakko.haverinen@uef.fi (J.H.)

**Keywords:** evolution of tetrodotoxin sensitivity, vertebrate animals, cardiac sodium current

## Abstract

Evolutionary origin and physiological significance of the tetrodotoxin (TTX) resistance of the vertebrate cardiac Na^+^ current (I_Na_) is still unresolved. To this end, TTX sensitivity of the cardiac I_Na_ was examined in cardiac myocytes of a cyclostome (lamprey), three teleost fishes (crucian carp, burbot and rainbow trout), a clawed frog, a snake (viper) and a bird (quail). In lamprey, teleost fishes, frog and bird the cardiac I_Na_ was highly TTX-sensitive with EC_50_-values between 1.4 and 6.6 nmol·L^−1^. In the snake heart, about 80% of the I_Na_ was TTX-resistant with EC_50_ value of 0.65 μmol·L^−1^, the rest being TTX-sensitive (EC_50_ = 0.5 nmol·L^−1^). Although TTX-resistance of the cardiac I_Na_ appears to be limited to mammals and reptiles, the presence of TTX-resistant isoform of Na^+^ channel in the lamprey heart suggest an early evolutionary origin of the TTX-resistance, perhaps in the common ancestor of all vertebrates.

## 1. Introduction

Opening of the voltage-sensitive Na^+^ channels (Na_v_) depolarizes plasma membrane and causes a rapid impulse spread in excitable cells via abrupt Na^+^ influx. The family of Na_v_1 channels consists of 9 α-subunits in mammals and 8 α-subunits in teleost fish, which arose from 4 ancestral genes of early vertebrates through individual gene duplications and whole genome duplication (3R), respectively [1–5]. On the basis of their affinity to guanidium compounds tetrodotoxin (TTX) and saxitoxin (STX), Na_v_ are classified into TTX-sensitive or TTX-resistant phenotypes. In most cases, TTX-resistance is achieved by a mutation of an aromatic amino acid (tyrosine, phenylalanine, tryptophan) in the position 401 (sequence numbering as in rat Na_v_1.4) to serine, cysteine or asparagine [6–8]. However, other amino acid substitutions in various sites of the Na^+^ channel pore have been reported in different TTX carrying vertebrates or their predators involving several Na_v_1 genes [9–12].

Resistance to TTX has independently arisen in several vertebrate groups, probably in response to feeding on organisms that contain guanidium toxins. Although the probability of dietary exposure to TTX or STX is low in mammals, three TTX-resistant Na^+^ channel isoforms are expressed either in the heart (Na_v_1.5) or in the peripheral nervous system (Na_v_1.8, Na_v_1.9) [6,13]. Considering that selection pressure on I_Na_ via guanidium toxin exposure is absent in most mammals, physiological significance and evolutionary origin of TTX-resistance of the mammalian I_Na_ remains an enigma. It is possible that TTX-resistant isoforms are evolutionary vestiges from their predecessors, which needed them to avoid guanidium poisoning. Consistently with this hypothesis, it has been suggested that TTX-resistance of the cardiac Na^+^ channel appeared in early vertebrates before the divergence of fish and mammalian lineages [7]. The vertebrate cardiac Na_v_1.5 is assumed to be an ortholog of the ancestral TTX-resistant channel from which Na_v_1.8 and Na_v_1.9 were derived by two successive gene duplications later in tetrapod evolution [14]. While synteny of the three genes strongly suggests that Na_v_1.8 and Na_v_1.9 arose from Na_v_1.5 by two successive gene duplications, there is little evidence that the ancestral vertebrate gene was TTX-resistant. If Na_v_1.5 represents an ancestral TTX-resistant gene, I_Na_ is expected to be TTX-resistant not only in mammalian heart but also in the hearts of “lower” vertebrates, including reptiles, frogs and fishes (provided that Na_v_1.5 is expressed in those hearts). Several preliminary findings suggest, however, that I_Na_ of frog and fish hearts might be more sensitive to TTX than the mammalian cardiac I_Na_ [15–18], although an opposite view for the fish has also been presented [7]. However, the TTX-phenotype of the vertebrate cardiac I_Na_ has, until now, remained unresolved in several major vertebrate groups.

To better understand the underlying reasons and physiological significance of the TTX-resistance of the mammalian cardiac I_Na_, evolution of TTX-resistance of the cardiac I_Na_ needs to be known. Therefore, this study aims to examine the phenotype of cardiac I_Na_ in regard to its blocking sensitivity by TTX in three main classes of ectothermic vertebrates (fishes, frogs and reptiles). Patch-clamp method on enzymatically isolated cardiac myocytes was used for direct determination of I_Na_ sensitivity to TTX. Some molecular data is also provided about the Na^+^ channel composition of vertebrate hearts.

## 2. Results and Discussion

### 2.1. TTX-Sensitivity of the Vertebrate Cardiac I_Na_

**Figure 1:**
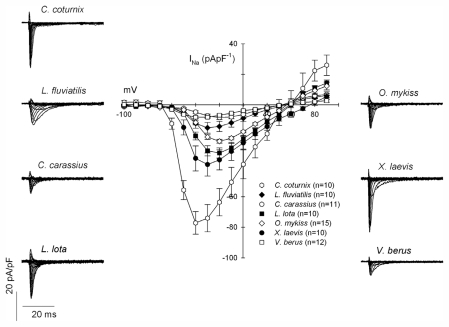
Density and voltage-dependence of cardiac I_Na_ in ventricular myocytes of 7 vertebrate species (*Lampetra fluviatilis*, *Carassius carassius*, *Lota lota*, *Oncorhynchus mykiss*, *Xenopus laevis*, *Vipera berus* and *Coturnix coturnix*). Experiments were conducted at 4 °C for the fish species and at 22 °C for the clawed frog (*X. laevis*), the European common viper (*V. berus*) and the quail (*Coturnix coturnix*). The results are means ± SEM of 5–15 myocytes from 3–8 animals for other species except for *V. berus* and *C. coturnix* (one animal).

**Figure 2:**
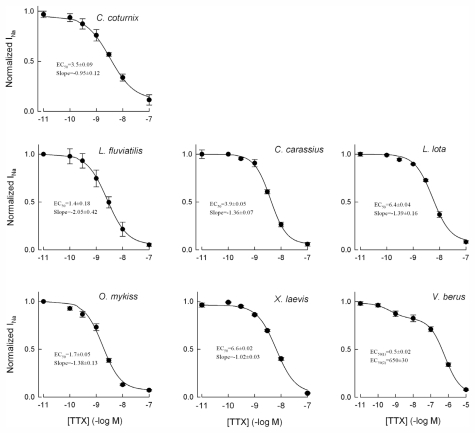
Concentration-response curves of TTX inhibition of the cardiac I_Na_ in ventricular myocytes of 7 vertebrate species (*Lampetra fluviatilis*, *Carassius carassius*, *Lota lota*, *Oncorhynchus mykiss*, *Xenopus laevis*, *Vipera berus and Coturnic coturnix*). Concentration for half-maximal inhibition of I_Na_ (EC_50_ in nmol·L^−1^) and Hill slope factor are also shown for each curve. The results are means ± SEM of 5–12 myocytes 3–8 animals for other species except for *V. berus* and *C. coturnix* (one animal). Note the different x-axis scale for *Vipera berus*.

**Figure 3:**
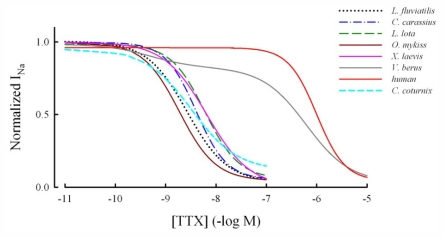
Comparison of TTX sensitivity of cardiac I_Na_ in 7 vertebrate animals (*Lampetra fluviatilis*, *Carassius carassius*, *Lota lota*, *Oncorhynchus mykiss*, *Xenopus laevis*, *Vipera berus* and *Coturnix coturnix*) as concentration-response curves. The data for the human cardiac I_Na_ are from [6].

TTX sensitivity of the ventricular I_Na_ was determined in four fish species including the evolutionary old cyclostome, the lamprey (*Lampetra fluviatilis)*, a frog (*Xenopus laevis*), a reptile (common European viper, *Vipera berus*) and a bird (quail, *Coturnix coturnix*). Voltage-dependence of I_Na_ was similar in all ectothermic vertebrates with the peak I_Na_ at −20~−40 mV, but the current density strongly varied between species (Figure 1). Hill slopes of the concentration-response curve were −0.95~−1.39 in the three teleost species and the frog, while the slope of the lamprey I_Na_ was shallower (−2.05) (Figure 2). The concentration-response curve of the *V. berus* I_Na_ was best fit by assuming two competitive binding sites. There were little differences in TTX sensitivity of I_Na_ among the fishes and the frog; in all species I_Na_ was highly sensitive to TTX with EC_50_-values between 1.4 (*L. fluviatilis*) and 6.6 nmol·L^−1^ (*X. laevis*). When these values are compared to the EC_50_ value of the mammalian cardiac I_Na_ (1 μmol·L^−1^), the I_Na_ of the ectotherms is 2–3 orders of magnitude more sensitive than the I_Na_ of the mammalian heart (Figure 3); I_Na_ of *L. fluviatilis* and *X. laevis* are 714 and 152 times more sensitive to TTX than the I_Na_ of the human heart. A clear exception among the ectothermic vertebrates is the snake, *V. berus*, with two apparent binding sites of I_Na_ inhibition: a high affinity inhibition with EC_50_ of 0.5 ± 0.2 nmol·L^−1^ and a low affinity inhibition with EC_50_ of 0.65 ± 0.03 μmol·L^−1^. The low affinity component constituted 80 ± 0.3% of the total I_Na_ indicating that this current system is mainly TTX-resistant in the snake heart. Thus, our analysis suggests that cardiac I_Na_ of the ectothermic vertebrates is highly TTX-sensitive in fishes (including the lamprey) and frogs, but TTX-resistant in reptiles. Interestingly, the I_Na_ of the bird (*C. coturnix*) heart was highly sensitive to TTX with EC_50_ value of 3.5 ± 0.09 nmol·L^−1^. Accordingly, among homeotherms, the avian heart seems to be TTX-sensitive, while the mammalian heart is TTX-resistant.

The present electrophysiological findings on TTX sensitivity of the cardiac I_Na_ are consistent with the [^3^H]STX binding experiments on vertebrate cardiac membranes indicating a high affinity binding (K_d_ = 5.1 nM) in the frog (*Rana pipiens*) and a low affinity binding in the turtle (*Chrysemys scripta elegans*) (K_d_ = 2.3 μM) [19]. The mammalian cardiac I_Na_ is well-known for its TTX resistance (EC_50_ about 1 μM) [6,20,21], while results on the TTX sensitivity of the avian cardiac I_Na_ are controversial. Indirect estimations of I_Na_ from the velocity of action potential upstroke give EC_50_ values for TTX of the chick heart in the range of 10–20 nM suggesting that the avian cardiac I_Na_ is TTX-sensitive [22,23]. On the other hand, [^3^H]TTX or [^3^H]STX binding experiments suggest that, similar to the mammalian cardiac Na^+^ channels, the chick cardiac Na^+^ channels have low affinity to guanidium toxins [19,24]. Our direct patch-clamp studies on the TTX sensitivity of the quail ventricular I_Na_ are consistent with the electrophysiological studies on the chick heart. Therefore, the current state of knowledge suggests that the I_Na_ of mammalian and reptilian hearts is TTX-resistant with EC_50_ in the micromolar range, while the I_Na_ of piscine, amphibian and avian hearts is 2–3 orders of magnitude more sensitive to TTX, *i.e.*, in the range of 1.4–6.6 nmoles·L^−1^ (Figure 3).

The high TTX sensitivity of the cardiac I_Na_ in the evolutionally old cyclostome vertebrate, the three teleost species, the frog and the bird suggests that TTX resistance is largely limited to mammals and reptiles. This division of vertebrate hearts in TTX-resistant and TTX-sensitive forms might suggest that the evolution of TTX-resistance of the cardiac I_Na_ appeared some 340 million years ago in the common ancestor of the Amniote tetrapods and the trait was secondarily lost in the bird lineage. Furthermore, the present findings on TTX phenotype of the vertebrate cardiac I_Na_ are apparently inconsistent with the suggestion that TTX resistance appeared before the divergence of the fish and mammalian lineages, because I_Na_ of fish and frog hearts appears to be TTX-sensitive (but see Section 2.3).

### 2.2. Alpha Subunit Composition of the Cardiac Na^+^ Channels

**Figure 4:**
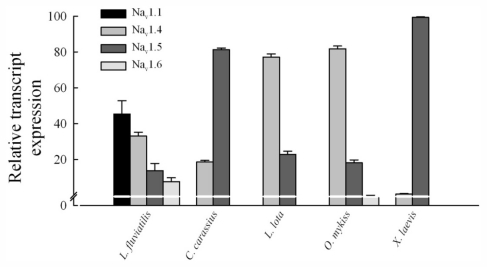
Expression pattern of Na^+^ channel isoforms in the heart of 5 vertebrate species (*Lampetra fluviatilis*, *Carassius carassius*, *Lota lota*, *Oncorhynchus mykiss* and *Xenopus laevis*). Relative transcript expression of different Na^+^ channel isoforms are shown as means ± SEM (*n* = 2–5 animals).

The above conclusions on TTX-sensitivity/resistance are solely based on the phenotype of cardiac I_Na_ in enzymatically isolated cardiac myocytes, without taking into account the molecular basis of the I_Na_. The high TTX-sensitivity of piscine, amphibian and avian cardiac I_Na_ raises a question about the Na^+^ channel composition of their hearts. TTX-resistance of the mammalian cardiac I_Na_ is due to the predominance of the TTX-resistant Na^+^ channel isoform, Na_v_1.5. To find out the molecular basis for TTX-sensitivity of the cardiac I_Na_ in ectothermic vertebrates, Na^+^ channel composition was examined by molecular cloning and quantitative PCR from fish and frog hearts. In addition to previously sequenced trout genes [18], partial cDNA sequences for Na_v_1.4 and Na_v_1.5 were cloned from burbot (658 bp and 662 bp, respectively), crucian carp (592 and 668 bp), lamprey (1797 and 2082 bp) and frog (749 and 656 bp). Additionally, partial sequences of putative Na_v_1.1 (2262 bp) and Na_v_1.6 (644 bp) channels were cloned from the lamprey. Surprisingly, in the *Xenopus* heart Na_v_1.5 was almost an exclusive alpha subunit isoform constituting over 99% the ventricular transcripts (Figure 4). Similarly, in crucian carp Na_v_1.5 formed 82% of the cardiac Na^+^ channel transcripts, the rest being represented by Na_v_1.4. Thus, in spite of the high TTX-sensitivity of the I_Na_, the predominant cardiac isoform in these ectotherms was Na_v_1.5. Interestingly, in burbot and rainbow trout ventricles Na_v_1.4 was clearly the dominant isoform forming 67% and 72% of the ventricular transcripts, respectively (Na_v_1.5 represented only 32% and 28% of the transcripts). Isoform composition of the lamprey heart was clearly different from those of other ectotherms. Na_v_1.1 seemed to be the most abundant isoform at the transcript level (45%), followed by Na_v_1.4 (33%), Na_v_1.5 (13%) and Na_v_1.6 (9%). These comparisons show that alpha subunit composition of the cardiac Na^+^ channel is variable among ectothermic vertebrates with the predominance of Na_v_1.4 and Na_v_1.5 isoforms in other ectotherms except the lamprey.

In mammals, the low TTX sensitivity of the cardiac I_Na_ is based on almost exclusive expression of the Na_v_1.5 in the heart; over 90% of the mammalian cardiac Na^+^ channels are Na_v_1.5, with the exception of mouse heart (70%) [25]. It is noteworthy is that the TTX resistance/sensitivity of the piscine and amphibian I_Na_ is not tightly associated with any particular Na^+^ channel isoform. Even though Na_v_1.5 is the main Na^+^ channel isoform (>80%) in the hearts of crucian carp and *Xenopus*, the cardiac I_Na_ is highly sensitive to TTX in both species. In burbot and trout, Na_v_1.4 forms about 80% of the cardiac Na^+^ channels transcripts and, as expected on the basis of high TTX sensitivity of this isoform in mammals, the cardiac I_Na_ in both fish species is highly TTX-sensitive. The TTX-sensitive I_Na_ of the lamprey heart is based on expression of four different Na^+^ channels. Sequence comparisons tentatively suggest that these channels are orthologs to Na_v_1.1, Na_v_1.4, Na_v_1.5 and Na_v_1.6 of the higher vertebrates. This may signify that lamprey heart expresses all four Na^+^ channels of the “basal” vertebrate which appeared after the two rounds of whole genome duplication (2R) [26,27].

### 2.3. Amino Acid Sequence of the TTX Binding Site in Domain I

**Figure 5:**
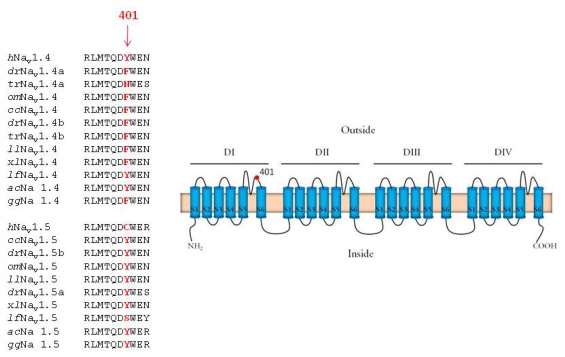
Sequence alignment of the TTX binding site in the pore loop region between S5 and S6 of the domain I in Na^+^ channel isoforms Na_v_1.4 and Na_v_1.5 from *Lampetra fluviatilis* (*lf*), *Carassius carassius* (*cc*), *Lota lota* (*ll*), *Oncorhynchus mykiss* (*om*), *Danio rerio* (*dr*, ABB29445 for Nav1.4a and ABB29446 for Nav1.4b), *Tagifugu rubribes* (*tr*, ABB29441 for Nav1.4a and ABB29442 for Nav1.4b), *Xenopus laevis* (*xl*), *Anolis carolinens* (*ac*, BK007956 for Na_v_1.4 and BK007957 for Na_v_1.5), *Gallus gallus* (*gg*, BK007949 for Na_v_1.4 and XM_418535 for Na_v_1.5) and human (*h*, NP_000325). The critical amino residues for TTX-sensitivity in the position 401 (sequence numbering as in the rat isoform Na_v_1.4) are shown in bold red.

That the phenotype of the cardiac I_Na_ in several ectothermic vertebrates is highly TTX-sensitive irrespective of the Na^+^ channel isoform raises a question about the amino acid composition of the TTX binding site, in particular in the position 401 of the Na_v_1.5. Consistent with the TTX-sensitive electrophysiological phenotype, Na_v_1.5 of the frog and three teleost fish species (crucian carp, burbot, trout) had an aromatic amino acid (tyrosine) in the position of 401 probably rendering the channel highly sensitive to TTX (Figure 5). The same applies also to the Na_v_1.5 of the zebra fish, contrary to the suggestion of Soong and Venkatesh [7]. Interestingly, the putative Na_v_1.5 ortholog of the lamprey had a non-aromatic amino acid (serine) in this position, which is known to confer TTX-resistance to the I_Na_. While the low expression level (13%) of the Na_v_1.5 may explain why I_Na_ in the lamprey heart is TTX-sensitive, the presence of serine in the critical position of the lamprey Na_v_1.5 violates the assumption that TTX-resistant Na^+^ channel first appeared in the common ancestor of the amniote vertebrates (see Section 2.1). Isoform composition and TTX binding site was not examined from the snake and the quail hearts. However, the aromatic amino acid (tyrosine) in the position 401 of the chicken (*Gallus gallus*) Na_v_1.5 is completely in line with the TTX-sensitivity of the bird cardiac I_Na_. In contrast, Y401 of the Na_v_1.5 in green anole (*Anolis carolinensis*) contradicts the finding that large portion of the cardiac I_Na_ of the snake heart is TTX-resistant [4]. The presence of TTX-sensitive and TTX-resistant components in the cardiac I_Na_ of the snake heart may suggest that there exists variability in TTX-sensitivity of I_Na_ among different reptilian lineages. Therefore, more reptilian species including representatives of snakes, lizards, turtles, crocodiles and birds should be examined in regard to cardiac Na^+^ channel composition and their TTX-sensitivity.

## 3. Conclusions

The present electrophysiological findings suggest that the cardiac phenotype of I_Na_ is TTX-sensitive in fishes, frogs and birds, and TTX-resistant in mammals and at least in some reptiles. Furthermore, it appears that the expression of Na_v_1.5 as the major cardiac Na^+^ channel does not automatically mean a TTX-resistant I_Na_ phenotype: in fishes, frogs and birds Na_v_1.5 has an aromatic amino acid in the position 401 and therefore the cardiac I_Na_ is highly sensitive to TTX.

**Figure 6:**
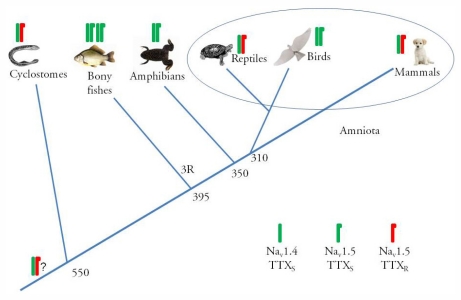
A scheme showing putative evolutionary origin for TTX resistance of cardiac I_Na_ in vertebrate animals. The scheme is based on patch-clamp analysis of cardiac I_Na_ in isolated cardiac myocytes. The presence of TTX-sensitive (green) or TTX-resistant (red) forms of the major cardiac Na^+^ channel alpha subunits, Na_v_1.4 and Na_v_1.5, are indicated. Na^+^ channel is assumed to be TTX-sensitive if there is an aromatic amino acid (tyrosine, phenylalanine, tryptophan) in the site 401.

The presence of S401 in the Na_v_1.5 ortholog of the lamprey is consistent with the hypothesis that TTX-resistance appeared early in the vertebrate evolution, possibly in the common ancestor of all vertebrates (Figure 6) [3]. The wide-spread occurrence of the TTX-sensitive isoform of Na_v_1.5 in fishes, frogs and birds suggests a later mutation of the channel, possibly due to functional requirements, turned the current TTX-sensitive. The reason why the mammalian heart and at least some reptile hearts express TTX-resistant I_Na_, while one of the amniote groups (birds) has a TTX-sensitive isoform still remains open. TTX-resistance of the cardiac I_Na_ in mammals and most reptiles is unlikely to be a defense mechanism against poisonous dietary items, since the whole-organism resistance to TTX requires that all members of the entire Na^+^ channel family have developed resistance to the toxin [28]. In mammals, only three members (Na_v_1.5, Na_v_1.8 and Na_v_1.9) of the Na_v_1 family are TTX-resistant. Therefore, it is more likely that the TTX-resistance of the cardiac Na_v_1.5 channel (and its paralogs) is a byproduct from a completely different selection pressure that resulted in mutation of the amino acid residue 401 of the domain I. In this scheme the whole-body resistance to TTX in pufferfishes (family Tetraodontidae), a goby fish (*Yongeichthys criniger*), a few amphibians and a garter snake (*Thamnopsis sirtalis*) appears as exceptions among the vertebrate animals [29,30]. These species accumulate TTX as a biologic defense agent against predators or as predators to prevent TTX intoxication from ingestion of TTX-bearing prey. A more detailed characterization of the cardiac I_Na_ phenotype and its molecular basis in different vertebrate groups is needed for deeper understanding of the evolution of cardiac I_Na_. Functional comparison of TTX-sensitive and TTX-resistant isoforms of bird and mammalian Na_v_1.5, respectively, might shed new light into this issue.

## 4. Experimental Section

### 4.1. Animals

Four fish, one frog, one reptile and one bird species were included in the experiments. River lamprey (*Lampetra fluviatilis* L.; Cephalaspidomorphi; body mass 43 ± 7 g, *n* = 10), crucian carp (*Carassius carassius* L.; Osteichthyes; 48 ± 8 g, *n* = 10) and burbot (*Lota lota* L.; Osteichthyes; 226 ± 17 g, *n* = 9) were caught from the wild and acclimated in the laboratory at +4 °C for a minimum of 4 weeks before experiments. Rainbow trout (*Oncorhynchus mykiss*, Walbaum; Osteichthyes; 249 ± 20 g, *n* = 12) were obtained from a local fish farm and were also acclimated in the lab at +4 °C. Different fish species were maintained separately in 500-L stainless steel tanks with a continuous supply of aerated groundwater under a 12 h:12 h light:dark photoperiod. During that time the fish were fed commercial trout food (Biomar, Brande, Denmark) to satiation five times a week. Clawed frog (*Xenopus laevis*, Daudin; Amphibia; 32 ± 5 g, *n* = 8) were from the stock maintained and reproduced in our lab. Frogs were reared in 100-L aquaria at 20 ± 1 °C and fed *ad libitum* commercial frog food (Frog Brittle, Nasco, Fort Atkinson, WI, USA) every other day. Lighting of the aquaria was supplied by fluorescent lamps with a photoperiod of 12 h:12 h light:dark. A common European viper (*Vipera berus* L.; Reptilia; 440 g, *n* = 1) was caught from the wild in August and maintained in a glass terrarium at room temperature (20 °C). One quail (*Coturnix coturnix*; 382 g, *n* = 1) was used in patch-clamp studies. All experiments were conducted with the consent of the national committee for animal experimentation (permission STH252A).

### 4.2. Patch-Clamp Recording of I_Na_

Experiments were conducted on enzymatically isolated ventricular myocytes. Cardiac myocytes were isolated by retrograde perfusion of the heart with a trypsin and collagenase containing solution as described earlier [17]. Myocytes were used within 8 hours from isolation. A small aliquot of myocyte suspension was transferred to a recording chamber (RC-26, Warner Instrument Corp, Brunswick USA; volume 150 μL) and superfused with external saline solutions at the rate of 1.5–2.0 mL·min^−1^. Initially, the myocytes were superfused with normal K^+^-based saline (containing in mmol·L^−1^: 150 NaCl, 5.4 KCl, 1.8 CaCl_2_, 1.2 MgCl_2_, 10 glucose, 10 HEPES, 0.01 nifedipine, pH adjusted to 7.7 with NaOH) where gigaseal and whole-cell patch clamp mode of recording were established. Internal perfusion of the myocytes with pipette solution (containing in mmol·L^−1^: 5 NaCl, 130 CsCl, 1 MgCl_2_, 5 EGTA, 5 Mg_2_ATP, 5 HEPES, pH adjusted to 7.2 with CsOH) was continued in this external saline solution for about 3 min to allow buffering of intracellular Ca^2+^ with 5 mM EGTA. Then, the solution flow was switched to a low-Na^+^ external solution (containing in mmol·L^−1^: 20 NaCl, 120 CsCl, 1 MgCl_2_, 0.5 CaCl_2_, 10 glucose, 10 HEPES, 0.01 nifedipine, pH adjusted to 7.7 with CsOH) to reduce driving force for Na^+^ influx and improve reliable recording of I_Na_ [31]. Low external Na^+^ concentration (20 mM), low experimental temperature (4–22 °C) and relatively small size of the myocytes (17–94 pF) rendered I_Na_ small (<2 nA) enough for a good voltage control. To ensure undistorted recordings a minimum of 80% series resistance compensation (9.09 ± 0.56 MΩ before compensation, *n* = 82) was routinely applied. I_Na_ was recorded at the sampling rate of 10 kHz and the traces were low-pass filtered at 5 kHz. The calculated liquid-junction potential of the electrodes was about 1.5 mV which was not corrected in the results.

Concentration-dependence of I_Na_ inhibition was determined by exposing myocytes to cumulatively increasing concentrations of TTX (10^−10^–10^−7^ M; TTX citrate from Tocris Cookson). Cells were exposed to each TTX concentration for 5 min to reach a steady-state inhibition of peak I_Na_, elicited by a depolarizing pulse from −120 mV to −20 mV for every 5th second. Concentration-response curves were fitted with an equation for one binding site:

$$\text{I}={\text{I}}_{\text{min}}+\frac{{\text{I}}_{\text{max}}-{\text{I}}_{\text{min}}}{1+10^{(\text{log}\;\text{EC}50-C)\times H}}$$

or in the case of the snake I_Na_ with an equation for two competitive binding sites using SigmaPlot 11.0 software.

$$\text{I}={\text{I}}_{\text{min}}+\left({\text{I}}_{\text{max}}-{\text{I}}_{\text{min}}\right)\times \left(\frac{F_{1}}{1+10^{\left(C-\text{log}\;\text{EC}50_{1}\right)}}+\frac{1-F_{1}}{1+10^{\left(C-\text{log}\;\text{EC}50_{2}\right)}}\right)$$

I_min_ is the residual I_Na_ at the highest TTX concentration, I_max_ the maximum I_Na_ before drug addition, EC_50_ (or EC_50(1)_ and EC_50(2)_ for *V. berus*) is the drug concentration which causes half-maximal inhibition of I_Na_ on its binding site, *C* the molar concentration of TTX, and *H* the Hill slope factor. Only myocytes for which a complete dose-response curve for TTX was obtained are included in the results.

### 4.3. Cloning of TTX Binding Area of Cardiac Na^+^ Channels

Heart was quickly excised and immediately frozen in liquid nitrogen. Total RNA was extracted with Trizol Reagent (Invitrogen, Carlsbad, USA) according to manufacturer’s instructions. RNA was qualified by gel electrophoresis and quantified by UV spectrophotometer. First-strand cDNA was prepared from the total cardiac RNA treated with RQ1 RNase-Free DNase (Promega, Madison, Wisconsin, USA). Reverse transcription was performed by M-MuLV Reverse Transcriptase RNase H+ (Finnzymes, Espoo, Finland) using random hexamers. Partial cDNAs corresponding to Na_V_1.4a, Na_V_1.5a and Na_V_1.6a were obtained by PCR using degenerative primers, designed to the conserved regions of corresponding mammalian and fish genes (Table 1). PCR was performed in a 25 μL reaction mixture containing 50 mM Tris·HCl, 1.5 mM MgCl_2_, 15 mM (NH_4_)_2_SO_4_, 0.1% Triton X-100, 200 μM of each dNTP, 1 U DyNAzyme EXT (Finnzymes), 2 μL cDNA and 5 pmol each primer. Amplification was performed in PCR conditions: initial denaturation at 94 °C for 2 min followed by 1–4 cycles with low annealing temperature at 94 °C for 30 s, 40 °C for 30 s and 72 °C for 90 s further followed by 31 cycles with higher annealing temperature at 55 °C and final extension at 72 °C for 5 min. PCR products were checked on a 0.8% agarose gel and if no products were obtained, 0.5 μL of the PCR product was reamplified using PCR conditions: initial denaturation at 96 °C for 2 min followed by 35 cycles at 95 °C for 30 s, 55 °C for 30 s and at 72 °C for 90 s, and final extension at 72 °C for 5 min. New primers were designed on the basis of the sequences obtained (Table 1).

After amplification, all PCR products were analyzed by gel electrophoresis, extracted from gel by Qiaex II gel extraction kit (QIAGEN, Valencia, California, USA), cloned into the pGEM-T Easy Vector (Promega) and sequenced by ABI Prism 310 Genetic Analyzer (Applied Biosystems, Foster City, California, USA). The partial Na^+^ channel sequences were assembled and analyzed using AutoAssembler software. Transeq software was used to convert the cDNA sequences into amino acid sequences.

### 4.4. Quantitative RT-PCR

Quantitative PCR was performed with DyNAmo HS SYBR Green qPCR Kit (Finnzymes) and Chromo4 Continuous Fluorescence Detector (MJ Research, Waltham, Massachusetts, USA) using primer pairs listed in Table 2. Primers for each Na^+^ channel gene were designed on the basis of the cloned sequences using Primer3-software (http://frodo.wi.mit.edu/primer3/) with options of 95–105 bp for product length and 57 °C for Tm. Specificity of each primer pair was checked using the plasmid DNA of the respective channel clone as template in RT PCR and with the melting curve analysis. The efficiency of the reaction was regarded acceptable if it was in the range of 3.2–3.6. A sample of total RNA (2 μg), extracted from ventricular tissue, was treated with RQ1 RNase-Free DNase (Promega), and the first strand cDNA synthesis was performed with random hexamers and M-MuLV RNase H+ (Finnzymes) at 25 °C for 10 min, at 37 °C for 30 min and at 85 °C for 5 min. Controls, in the absence of RT, were accomplished for each sample to determine possible DNA contamination. Polymerase was activated at 94 °C for 15 min and amplification was performed for 40 cycles at 94 °C for 10 s, 57 °C for 20 s and 72 °C for 30 s. After PCR, specificity of the reaction was checked by melting curve analysis. Two controls, one containing all the reaction components except the template and the other one containing the product of cDNA synthesis but without RT enzyme, was included in every experiment. DnaJA2 was used as a reference gene [32].

## Figures and Tables

**Table 1:** Primers used for the cloning of SCN genes from different species.

Target Gene	Primers for the First PCR	Primers for the Second PCR
**ccSCN4A**	F: ATGGCDMSCATKCTCCCTCC R: GCCATGGCNACCACVGCSAGGAT	F: ATGGCDMSCATKCTCCCTCC R: AGGTAGAARGAVCCCAGRAAKATGA
**ccSCN5A**	F: TCCTHCGRGACCCVTGGAAYTGGCT R: GCCATGGCNACCACVGCSAGGAT	F: TTCCTHCGRGACCCVTGGAAYTGGCT R: AGGTAGAARGAVCCCAGRAAKATGA
**lfSCN1A,** −**4A,** −**5A,** −**8A**	F: TCCTHCGRGACCCVTGGAAYTGGCT R: GCCATGGCNACCACVGCSAGGAT	F: TTCCTHCGRGACCCVTGGAAYTGGCT R: AGGTAGAARGAVCCCAGRAAKATGA
**lfSCN4A**	F: TGCTTGTCATCGCATGTTTT R: CCACATGGTCTCGATCCACT	F: TCATGACSCARGACTMCTGG R: CCACATGGTCTCGATCCACT
**lfSCN5A**	F: CCTTAAAACGTTCCGAGTGC R: CCACATGGTCTCGATCCACT	F: TCATGACSCARGACTMCTGG R: CCACATGGTCTCGATCCACT
**llSCN4A** **llSCN5A**	F: TCCTHCGRGACCCVTGGAAYTGGCT R: GCCATGGCNACCACVGCSAGGAT	F: TTCCTHCGRGACCCVTGGAAYTGGCT R: AGGTAGAARGAVCCCAGRAAKATGA
**xlSCN4A** **xlSCN5A**	F: TCCTHCGRGACCCVTGGAAYTGGCT R: GCCATGGCNACCACVGCSAGGAT	F: TTCCTHCGRGACCCVTGGAAYTGGCT R: AGGTAGAARGAVCCCAGRAAKATGA

**Table 2:** Primers used for quantitative PCR.

Target Gene	Primer Sequences	Amplicon Size (bp)
**ccSCN4A**	F: CAACAGTGATAACCTGACCA R: GGTTCGCTGGGTTTTCAATA	102
**ccSCN5A**	F: CCTTCAGACAACAGCAGCAC R: CTTGGCTCCTTCCACTTTGA	105
**ccDnaJA2**	F: GGACTTGTACGACCGTTATGG R:CGCCAAAGATATGGGAAAAGAT	93
**lfSCN1A**	F: AGTTCAACGAGACCGTCGAG R: CCACACAGGAGAGCATCCTT	100
**lfSCN4A**	F: TGCTTGTCATCGCATGTTTT R: CCACGGAAATGGTTTTCAAC	98
**lfSCN5A**	F: CCTTAAAACGTTCCGAGTGC R: GAGCTTTTTCACCGACTGGA	99
**lfSCN8A**	F: CGCTACCAAACGTCTCGTTC R: CCCTTGACTTCCCAGACGTA	103
**lfDnaJA2**	F: AGCCCCATGGACATATTTGA R: GCCCACAGACAACTGGTGTA	96
**llSCN4A**	F: AACGACAGCCTTGGTGATGT R: TCTGGCCGTTTCGGTAGTAG	103
**llSCN5A**	F: AACGCCTCCTTCTACTGCAA R: AACGCATCCTTGGCTCCTT	101
**llDnaJA2**	F: CCCGAGAAGAAGGAGCTGTA R: CCGAAGATGTGGGAGAAGAT	101
**omSCN4A**	F: CAGCAACAGTACGTGGGACT R: CAGAAGCGTTTCCACAGAGA	101
**omSCN5A**	F: ACACCCTCAACACCAACACA R: CGCTCCCTCCACTTTGTAGT	104
**omSCN8A**	F: GGAGCTCCATGAACGACTTC R: CTTCCCTGCATCTTGTCCAT	101
**omDnaJA2**	F: TTGTAATGGAGAAGGTGAGG R: TGGGCCGCTCTCTTGTATGT	233
**xlSCN4A**	F: CAACAGTACCTTAAACGCCACA R: TAGGCATCCCAGTCAATGGT	105
**xlSCN5A**	F: ATTTCAATGCGACCCAGATG R: CCGTCCTTGTGGTAGATGTT	106
**xlDnaJA2**	F: GTCGCAATGGGAGAAGAAGA R: CTTGCTGAGCTGAAGTTTGG	101
